# Identification of a novel m6A-related lncRNA pair signature for predicting the prognosis of gastric cancer patients

**DOI:** 10.1186/s12876-022-02159-3

**Published:** 2022-02-21

**Authors:** Jun-Mei Wang, Xuan Li, Peng Yang, Wen-Bin Geng, Xiao-Yong Wang

**Affiliations:** 1 Department of Gastroenterology, The Affiliated Changzhou Second People’s Hospital of Nanjing Medical University, Changzhou, 213000 China; 2 Dalian Medical University, Dalian, 116044 China; 3 Department of Gastroenterology, The First Affiliated Hospital of Nanjing Medical University, Nanjing, 210000 China

**Keywords:** N6-methyladenosine, Gastric cancer, lncRNA, Immune cell infiltration, Epithelial mesenchymal cell transition

## Abstract

**Background:**

Accumulating studies have demonstrated that lncRNAs play vital roles in the prognosis of gastric cancer (GC); however, the prognostic value of N6-methyladenosine-related lncRNAs has not been fully reported in GC. This study aimed to construct and validate an m6A-related lncRNA pair signature (m6A-LPS) for predicting the prognosis of GC patients.

**Methods:**

GC cohort primary data were downloaded from The Cancer Genome Atlas. We analysed the coexpression of m6A regulators and lncRNAs to identify m6A-related lncRNAs. Based on cyclical single pairing along with a 0-or-1 matrix and least absolute shrinkage and selection operator-penalized regression analyses, we constructed a novel prognostic signature of m6A-related lncRNA pairs with no dependence upon specific lncRNA expression levels. All patients were divided into high-risk and low-risk group based on the median risk score. The predictive reliability was evaluated in the testing dataset and whole dataset with receiver operating characteristic (ROC) curve analysis. Gene set enrichment analysis was used to identify potential pathways.

**Results:**

Fourteen m6A-related lncRNA pairs consisting of 25 unique lncRNAs were used to construct the m6A-LPS. Kaplan–Meier analysis showed that the high-risk group had poor prognosis. The area under the curve for 5-year overall survival was 0.906, 0.827, and 0.882 in the training dataset, testing dataset, and whole dataset, respectively, meaning that the m6A-LPS was highly accurate in predicting GC patient prognosis. The m6A-LPS served as an independent prognostic factor for GC patients after adjusting for other clinical factors (*p* < 0.05). The m6A-LPS had more accuracy and a higher ROC value than other prognostic models for GC. Functional analysis revealed that high-risk group samples mainly showed enrichment of extracellular matrix receptor interactions and focal adhesion. Moreover, *N*-cadherin and vimentin, known biomarkers of epithelial–mesenchymal transition, were highly expressed in high-risk group samples. The immune infiltration analysis showed that resting dendritic cells, monocytes, and resting memory CD4 T cells were significantly positively related to the risk score. Thus, m6A-LPS reflected the infiltration of several types of immune cells.

**Conclusions:**

The signature established by pairing m6A-related lncRNAs regardless of expression levels showed high and independent clinical prediction value in GC patients.

## Introduction

Gastric cancer (GC) is a major global disease, and it is the fifth most common cancer and the fourth most lethal malignancy. There were more than one million new cases and an estimated 769,000 deaths in 2020 [1], and more than 40% of the new cases and deaths occurred in China [2, 3]. In addition, 80% of patients with GC are diagnosed at an advanced stage [4]. Notably, the 5-year mortality rate for advanced GC is between 30 and 50% [5]. Overall, the prognosis of GC is not very optimistic, and it is necessary to identify novel biomarkers to reliably predict the survival outcomes of GC patients.

Of the over 160 RNA post-transcriptional regulatory marks in multiple RNA species, N6-methyladenosine (m6A) is the most common form modification on mRNA in higher eukaryotes, and it plays a vital role in RNA splicing, export, stability and translation [6]. Recently, accumulating studies have revealed that m6A modification is involved in multiple processes of tumorigenesis [7–11], and m6A modification, which is a reversible and dynamic process, is regulated by m6A regulators, including “writers” (methyltransferases), “readers” (signal transducers) and “erasers” (demethylases) [12]. Writers, including METTL3, METTL16, KIAA1429, WTAP, RBM15, RBM15B, and ZC3H13, mediate the RNA methylation modification process. Erasers include FTO and ALKBH5, and mediate the RNA demethylation process. In addition, signal transducers, including YT521-B homology (YTH) domain family members (YTHDF1, YTHDF2, and YTHDF3), YTH domain-containing proteins (YTHDC1 and YTHDC2), heterogeneous nuclear ribonucleoproteins family members (HNRNP and HNRNPA2B1), and insulin-like growth factor 2 mRNA-binding proteins (IGF2BPs; including IGF2BP1, IGF2BP2, and IGF2BP3), affect the reading of RNA methylation information, translation, stability and degradation of downstream RNAs [4, 13, 14]. In summary, m6A RNA methylation has a significant impact on RNA production and metabolism and is involved in the pathogenesis of multiple diseases, including GC [15].

Long non-coding RNAs (lncRNAs) represent the largest group of non-coding RNAs produced from the genome [16], and they are more than 200 nucleotides in length. Accumulating evidence has revealed that various lncRNAs contribute to gene expression at both the post-transcriptional and transcriptional levels. Additionally, aberrant lncRNA expression is strongly related to multiple cancers [12, 17] and serves as a diagnostic and prognostic marker for tumours [18]. Furthermore, lncRNAs can direct the expression of genes related to the activation of immune cells, thus altering the immune microenvironment and further contributing to the malignant phenotypes of some cancers [17, 19]. m6A-related lncRNAs are potential biomarkers for predicting the overall survival (OS) of lower-grade glioma patients and might be novel therapeutic targets [12]. However, m6A-related lncRNA signatures in GC patients need further exploration.

Epithelial–mesenchymal transition (EMT) is a process that enables polarized epithelial cells to transition towards a mesenchymal phenotype with increased cellular motility, and EMT occurs in many types of cancers [20]. In GC, the loss of E-cadherin expression stimulates cell transformation into a more invasive and less differentiated state through the EMT process [21]. However, the association between m6A-related lncRNAs and EMT factors in GC is not entirely clear.

In the present study, we analysed the value of a m6A-related lncRNA pair signature (m6A-LPS) in predicting the OS of GC patients and further validated the m6A-LPS in the testing dataset and the whole dataset. Notably, m6A-LPS served as an independent prognostic marker for GC independent of other clinical variables. Additionally, we identified differences in the expression of EMT biomarkers and immune cell infiltration between the high-risk and low-risk groups.

## Materials and methods

### Data collection and preparation, correlation analysis and differential expression analysis

All data, including the RNA-seq reads per kilobase per million (FPKM) data and clinical information of GC samples, were downloaded from The Cancer Genome Atlas (TCGA) database. By using GTF file annotation, mRNAs and lncRNAs were distinguished. m6A-related lncRNAs were defined as those with Pearson correlation coefficient > 0.4 and *p* < 0.001. Additionally, differential expression analysis of m6A-related lncRNAs between normal and adjacent tissue was performed using the R package limma, including thresholds of |log fold change (FC)| > 1.5 and false discovery rate (FDR) < 0.05.

### lncRNA pairs

The differentially expressed m6A-related lncRNAs were cyclically single paired, and a lncRNA pair matrix was constructed. Briefly, if the expression level of the first lncRNA was higher than that of the second lncRNA, the expression was assigned as 1; otherwise, the output was 0. In addition, the lncRNA pair was identified as a valid match when the number of pairs with an expression quantity of 0 or 1 accounted for more than 20% of the total lncRNA pairs.

### Construction of a m6A-LPS and evaluation of the relative risk score

First, we utilized univariate survival analysis based on the Kaplan–Meier method with the log-rank test to identify prognostic m6A-related lncRNA pairs, and a *p* value < 0.05 was considered to indicate statistical significance. To avoid overfitting, least absolute shrinkage and selection operator (LASSO)-penalized regression analysis was used to construct the best model. The following formula was used to calculate the risk score of each GC patient.

$${\text{m6A}} - {\text{LPS}} = \left( {{\text{Expr}}_{{\text{genepair - 1}}} \times {\text{ Coef}}_{{\text{genepair - 1}}} } \right) + \left( {{\text{Expr}}_{{\text{genepair - 2}}} \times {\text{ Coef }} -_{{\text{genepair - 2}}} } \right) + \, \cdots \, + ({\text{Expr}}_{{\text{genepair - n}}} \, \times \,{\text{Coef}}_{{\text{genepair - n}}} ),$$

where “n” means the total number of lncRNA pairs included in the signature, “Expr” is the matrix value of the lncRNA (either 1 or 0), and “Coef” is the coefficient of the lncRNA pair estimated from the LASSO regression model. All of the GC patients were randomly divided into a training dataset and a testing dataset. Then, the patients were divided into a high-risk group and a low-risk group based on the median risk score. Kaplan–Meier analysis and ROC curve analysis were used to evaluate the OS prediction ability and prognostic accuracy of m6A-LPS in the training dataset, the testing dataset, and the whole dataset. The sensitivity and specificity of m6A-LPS for GC patients was compared with those of other clinicopathological characteristics using ROC curve analysis and decision curve analysis (DCA) [22].

### Validation of the model and predictive nomogram

The chi-square test was used to confirm the relationship between the m6A-LPS and clinicopathological characteristics, and univariate and multivariate Cox regression analyses were used to determine whether the m6A-LPS was an independent prognostic predictor. Kaplan–Meier analysis was used to confirm the predictive value of the risk score in different clinicopathological feature subgroups. Additionally, a nomogram was constructed by integrating the m6A-LPS and clinicopathological features to predict the 1-, 3-, and 5-year OS of GC patients.

### Investigation of tumour-infiltrating immune cells

We used CIBERSORT to analyse the relationship between the risk score and immune cells. The relationships were analysed by Spearman correlation analysis, and *p* < 0.05 was considered to indicate statistical significance. The procedure used the R ggplot 2 package.

### Gene set enrichment analysis (GSEA)

GSEA was used to quantify the underlying Kyoto Encyclopedia of Genes and Genomes (KEGG) pathways associated with the m6A-LPS, and *p* < 0.05 and FDR < 0.05 were used as the criteria to identify significant pathways.

### Statistical analysis

All primary data were downloaded from TCGA, and all statistical analyses were performed using R (version 4.0.4) and PERL (version 5.32.1). Survival differences were determined using Kaplan–Meier curve and log-rank test analyses, and the survival curves were plotted with the R package survmine. Multivariate analyses were conducted using the Cox proportional hazard regression model. Clinical data were analysed using the chi-square test or Fisher’s exact test. For all results, a *p* value < 0.05 was considered to indicate statistical significance.

## Result

### Identification of differentially expressed m6A-related lncRNAs

**Fig. 1:**
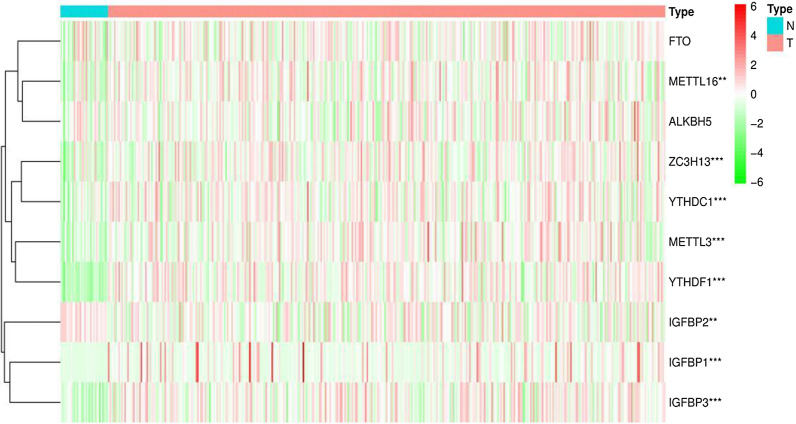
Expression of the 10 m6A regulators in GC patients. (***p < 0.001, **p < 0.01, *p < 0.05)

**Fig. 2:**
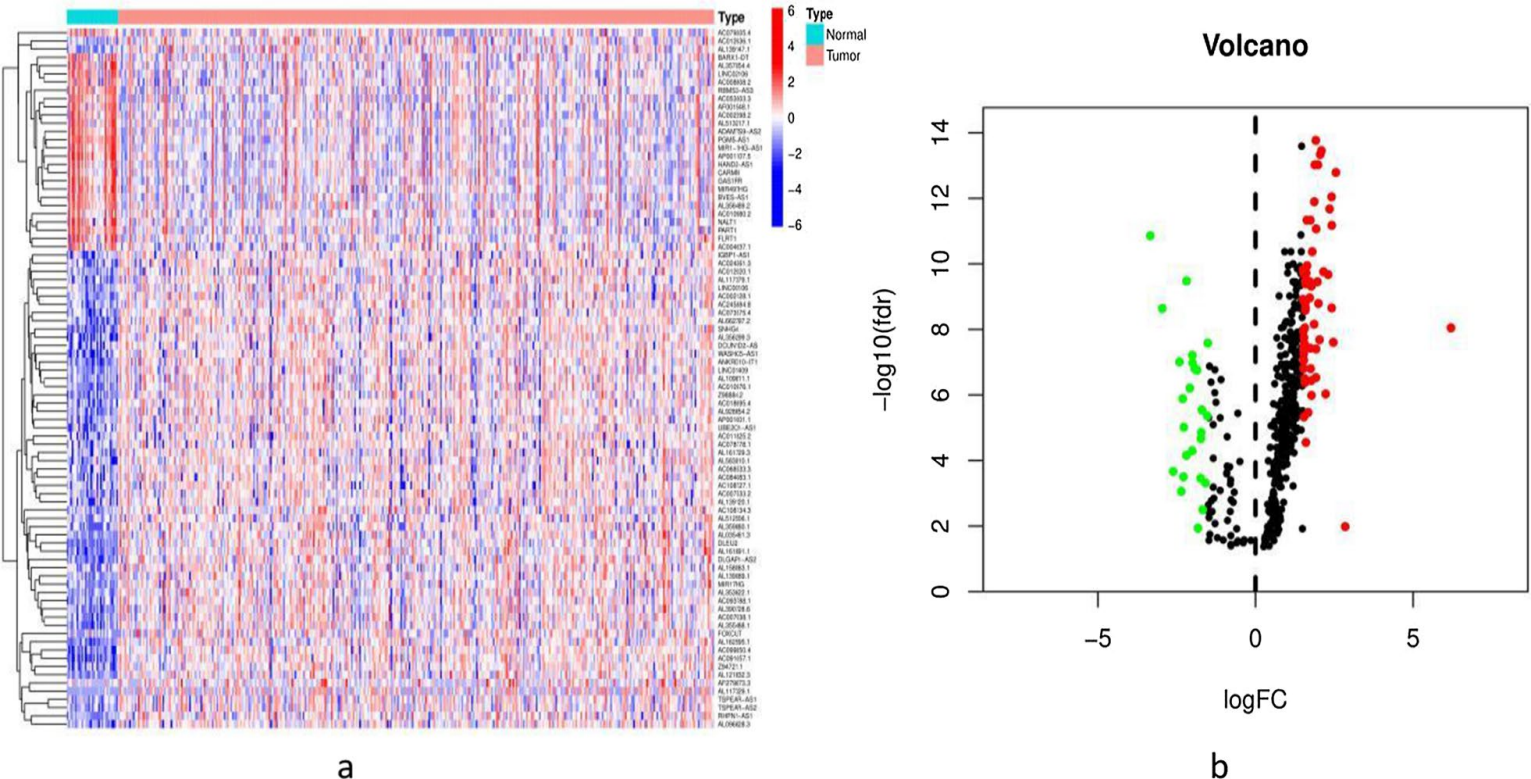
The 85 differently expressed lncRNAs in GC patients (**a** heatmap; **b** volcano plot)

The transcriptome profiling data of GC samples, including 32 adjacent and 375 tumour tissue samples, were downloaded from TCGA. We identified 14,086 lncRNAs in the GC dataset. A total of 23 m6A regulators were acquired from published studies (Table 1), and 10 of 23 m6A regulators with hazard ratio (HR) > 1 and *p* < 0.05 in GC patients were further screened in Kaplan–Meier Plotter (Table 2). Their expression in GC patients is shown in Fig. 1. Heatmap analysis showed that 10 m6A regulators were significantly more highly expressed in tumour tissue than in normal tissue (*p* < 0.05), except for FTO and ALKBH5. Furthermore, 491 lncRNAs related to 23 m6A regulators were identified, and 444 m6A-related lncRNAs were further selected based on 10 m6A regulators for the next part of the study. A total of 85 differentially expressed m6A-related lncRNAs were identified (Fig. 2a), with filter conditions of| log FC| > 1.5 and FDR < 0.05; of these, 60 were upregulated and 25 were downregulated (Fig. 2b).

**Table 1:** The 23 known m6A regulators

Writers	Readers	Erasers
METTL3, METTL16	YTHDF1, YTHDF2, YTHDF3	FTO
RBM15, RBM15B	HNRNPC, HNRNPA2B1	ALKBH5
ZC3H13, VIRMA	IGF2BP1, IGF2BP2, IGF2BP3	
KIAA1429	YTHDC1, YTHDC2	
WTAP	FMR1, LRPPRC, RBMX

**Table 2:** The 10 selected m6A regulators from Kaplan–Meier Plotter

m6A regulators	HR	*p*
IGF2BP1	1.49 (1.16–1.91)	0.0015
IGF2BP2	1.38 (1.16–1.64)	0.00023
IGF2BP3	1.58 (1.33–1.88)	1.1e−07
METTL3	1.81 (1.53–2.15)	4.8e−12
METTL16	1.28 (1.03–1.59)	0.027
YTHDC1	1.61 (1.34–1.94)	2.1e−07
YTHDF1	1.33 (1.1–1.61)	0.0037
ZC3H13	1.69 (1.42–2.01)	1.9e−09
FTO	1.81 (1.46–2.22)	1.3e−08
ALKBH5	1.4 (1.11–1.75)	0.0036

### Establishment of m6A-related lncRNA pairs and a risk assessment model

**Fig. 3:**
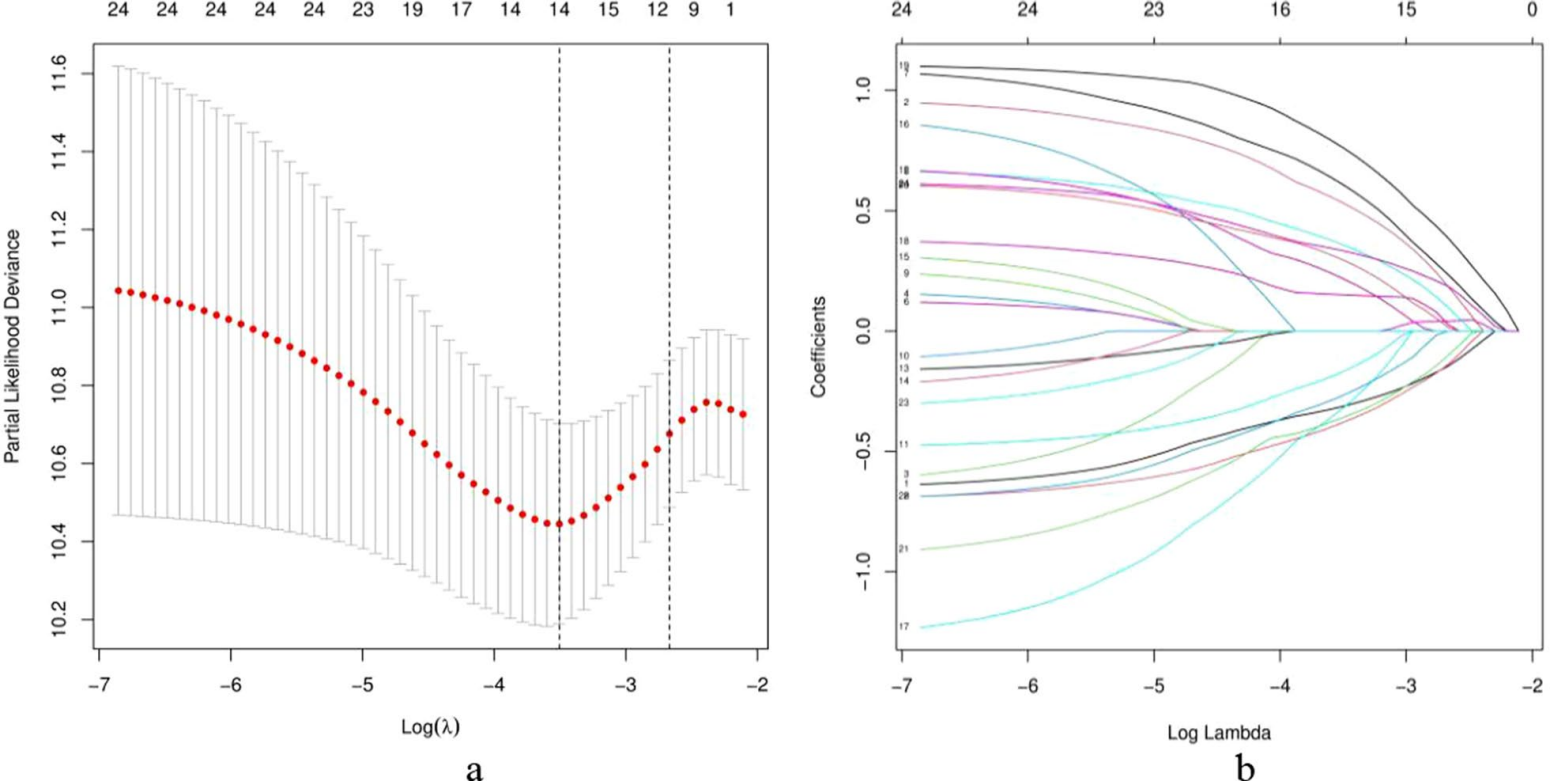
Characterization of the m6A-LPS. **a** LASSO coefficients of the 14 lncRNA pairs in GC patients. **b** Selection of the best parameters for GC patients on the basis of the LASSO model

First, among the 85 differentially expressed m6A-related lncRNAs, 2069 valid m6A-related lncRNA pairs were identified by using an iteration loop and a 0-or-1 matrix. We randomly divided 347 patients into a training dataset (N = 174) and a testing dataset (N = 173) (training dataset: test dataset = 1:1). We then used univariate Cox regression analysis and LASSO-penalized regression analysis to construct the m6A-LPS in the training dataset (Fig. 3). Finally, we identified 14 m6A-related lncRNA pairs and their corresponding coefficients (Table 3). The risk scores of each patient in the training dataset, testing dataset, and the whole dataset were calculated based on the following risk formula:

$$\begin{aligned} {\text{Riskscore}} & = \left( { - \,0.313282155226672*{\text{AC}}004637.1|{\text{AP}}001107.5} \right)\, \\ & \quad + \,\left( {0.533289565196042*{\text{AC}}010976.1|{\text{AC}}012020.1} \right) \\ & \quad + \,\left( {0.358601343882613*{\text{AC}}073575.4|{\text{LINC}}01409} \right) \\ & \quad + \,\left( {0.60937495396205*{\text{AC}}084083.1|{\text{IGBP1 - AS}}1} \right) \\ & \quad + \,\left( { - 0.38096026056395*{\text{AC}}091057.1|{\text{Z}}98884.2} \right) \\ & \quad + \,\left( { - 0.169592838571592*{\text{AL}}117379.1|{\text{AL}}662797.2} \right) \\ & \quad + \,\left( {0.218483698750099*{\text{AL}}121832.3|{\text{AL}}512506.1} \right) \\ & \quad + \,\left( { - 0.321964769101311*{\text{AL}}353622.1|{\text{PART}}1} \right) \\ & \quad + \,\left( {0.151028401592232*{\text{AL}}356489.2|{\text{IGBP1 - AS}}1} \right) \\ & \quad + \,\left( {0.762974482208771*{\text{AL}}356489.2|{\text{PART}}1} \right) \\ & \quad + \,\left( {0.296036005477854*{\text{AL}}357054.4|{\text{NALT}}1} \right) \\ & \quad + \,\left( { - 0.360994796555978*{\text{AL}}512506.1|{\text{MIR}}17{\text{HG}}} \right) \\ & \quad + \,\left( { - 0.268165795011373*{\text{AP}}001001.1|{\text{BVES - AS}}1} \right) \\ & \quad + \,\left( {0.333810275163147*{\text{CARMN}}|{\text{NALT}}1} \right). \\ \end{aligned}$$

**Table 3:** The 14 m6A-related lncRNA pairs in the prognostic signature

Signature pair	Gene A	Gene B	Coef
Pair 1	AC004637.1	AP001107.5	− 0.313282155226672
Pair 2	AC010976.1	AC012020.1	0.533289565196042
Pair 3	AC073575.4	LINC01409	0.358601343882613
Pair 4	AC084083.1	IGBP1-AS1	0.60937495396205
Pair 5	AC091057.1	Z98884.2	− 0.38096026056395
Pair 6	AL117379.1	AL662797.2	− 0.169592838571592
Pair 7	AL121832.3	AL512506.1	0.218483698750099
Pair 8	AL353622.1	PART1	− 0.321964769101311
Pair 9	AL356489.2	IGBP1-AS1	0.151028401592232
Pair 10	AL356489.2	PART1	0.762974482208771
Pair 11	AL357054.4	NALT1	0.296036005477854
Pair 12	AL512506.1	MIR17HG	− 0.360994796555978
Pair 13	AP001001.1	BVES-AS1	− 0.268165795011373
Pair 14	CARMN	NALT1	0.333810275163147

**Fig. 4:**
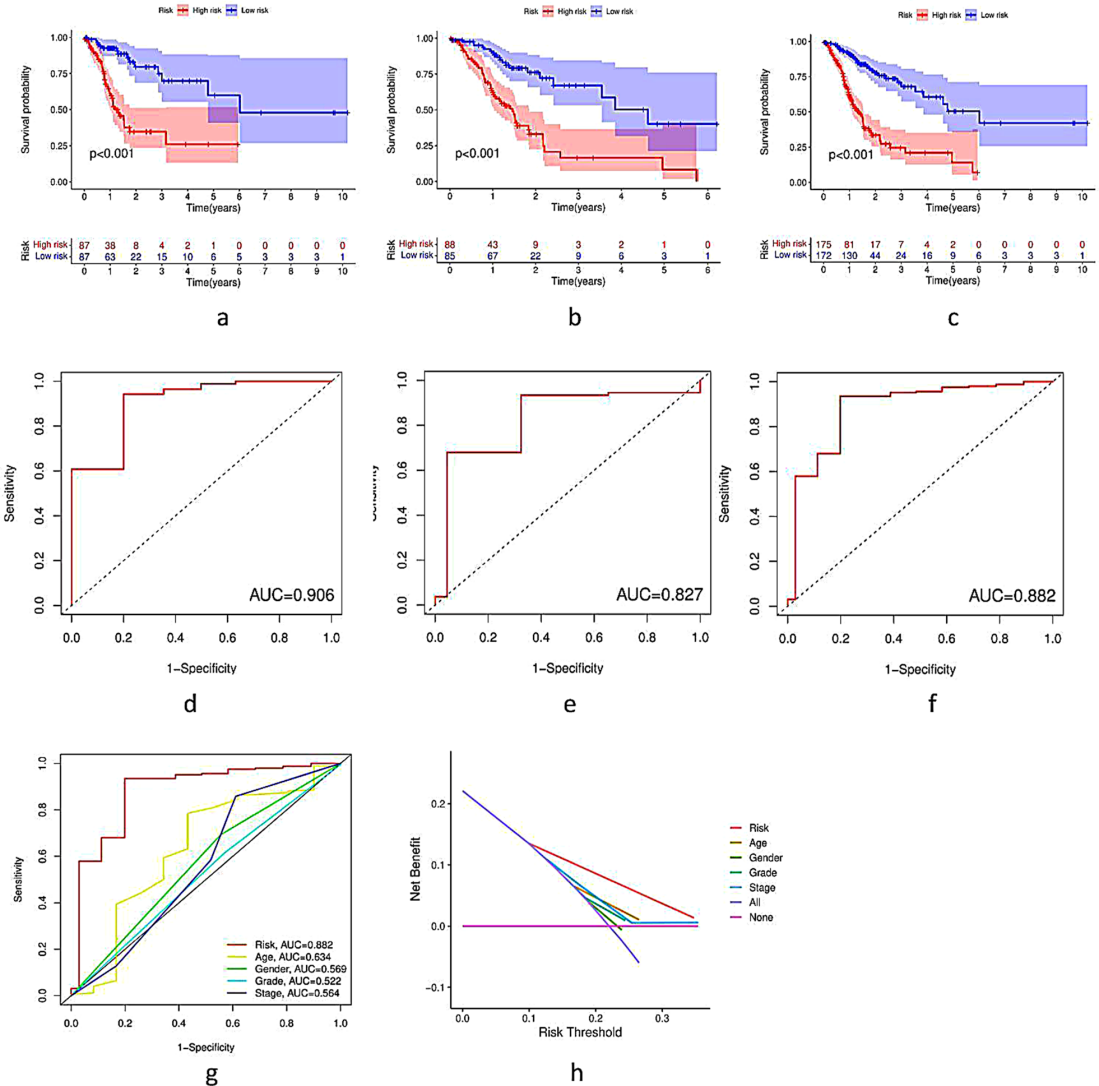
Kaplan–Meier survival curve (**a**–**c**) and ROC curve (**d**–**f**) analysis of the m6A-LPS between the high-risk group and low-risk group in the training dataset (**a**, **d**), testing dataset (**b**, **e**), and whole dataset (**c**, **f**). **g** Comparison of the 5-year ROC curves of the m6A-LPS and other clinicopathological features. **h** DCA of the risk factors

Patients in the three datasets were further divided into a high-risk group and a low-risk group based on the median risk score. The Kaplan–Meier curve analysis results showed that the low-risk group had a better prognosis than the high-risk group in the three datasets (*p* < 0.001) (Fig. 4a–c). Moreover, the area under the curve (AUC) for 5-year OS was 0.906, 0.827, and 0.882 in the training dataset, testing dataset, and whole dataset, respectively (Fig. 4d–f). Furthermore, the AUC of m6A-LPS was 0.882, exhibiting superior performance compared to traditional clinicopathological characteristics in predicting the prognosis of GC patients (Fig. 4g–h).

### Prognostic value of m6A-LPS and its relationship with clinicopathological features

**Fig. 5:**
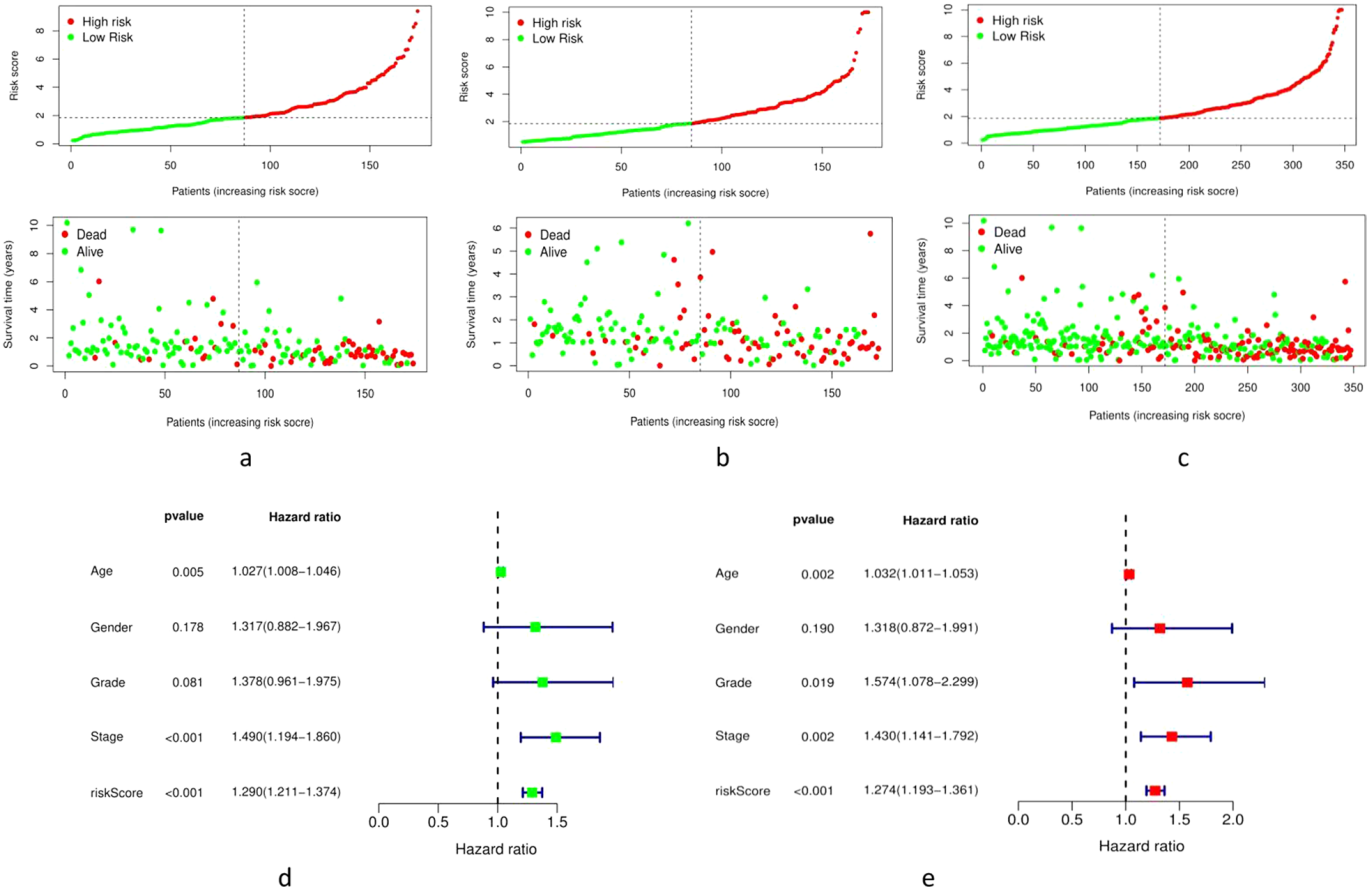
The distributions of the m6A-LPS along with the corresponding survival status based on the risk curve and the independence of the m6A-LPS in OS. (**a** the training dataset, **b** testing dataset, **c** whole dataset, **d** Univariate cox regression analysis, **e** Multivariate cox regression analysis)

**Fig. 6:**
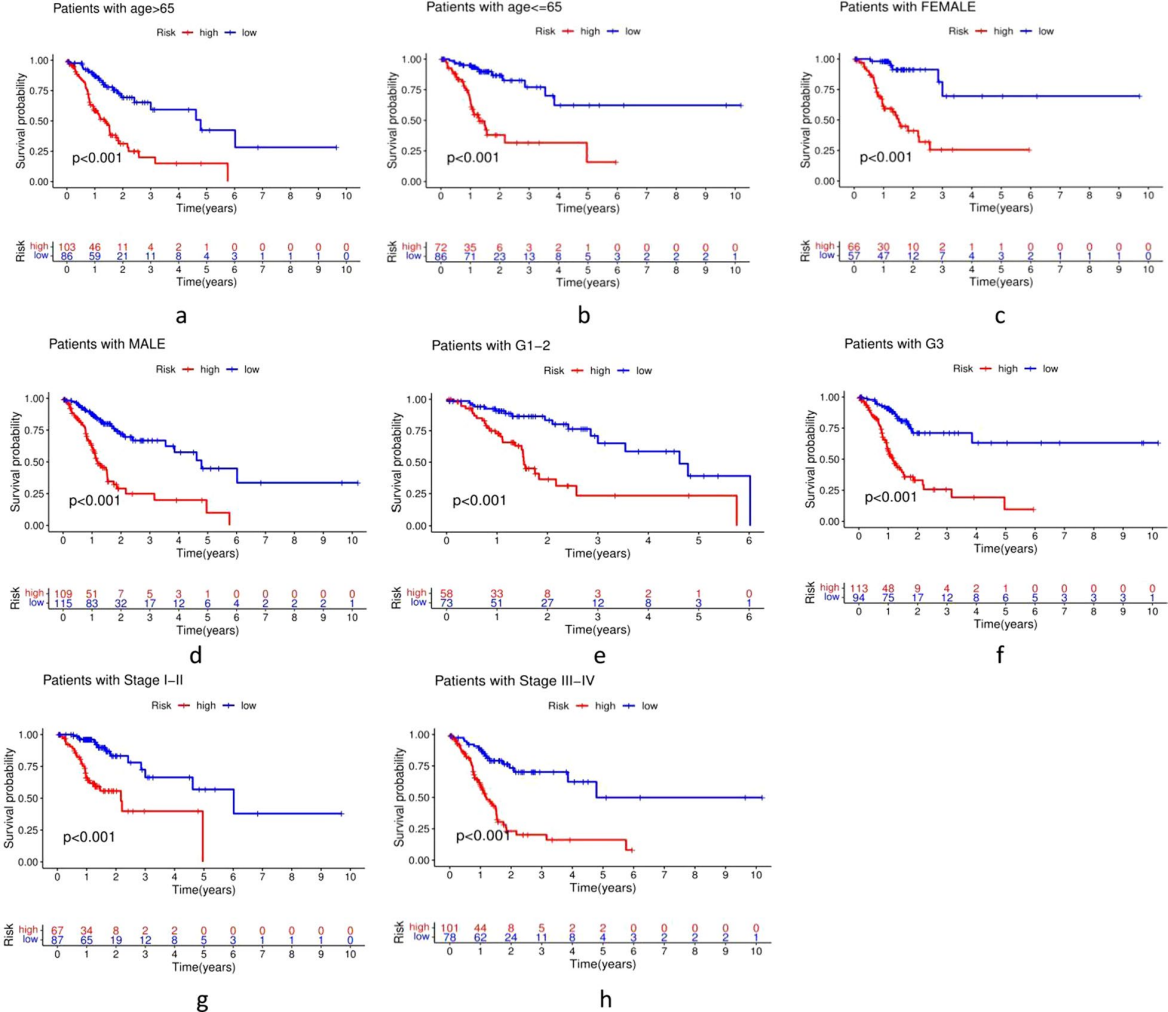
Kaplan–Meier survival curves for the high-risk and low-risk groups stratified by clinical factors including age (**a**, **b**), sex (**c**, **d**), grade (**e**, **f**), and stage (**g**, **h**) in the whole dataset

**Fig. 7:**
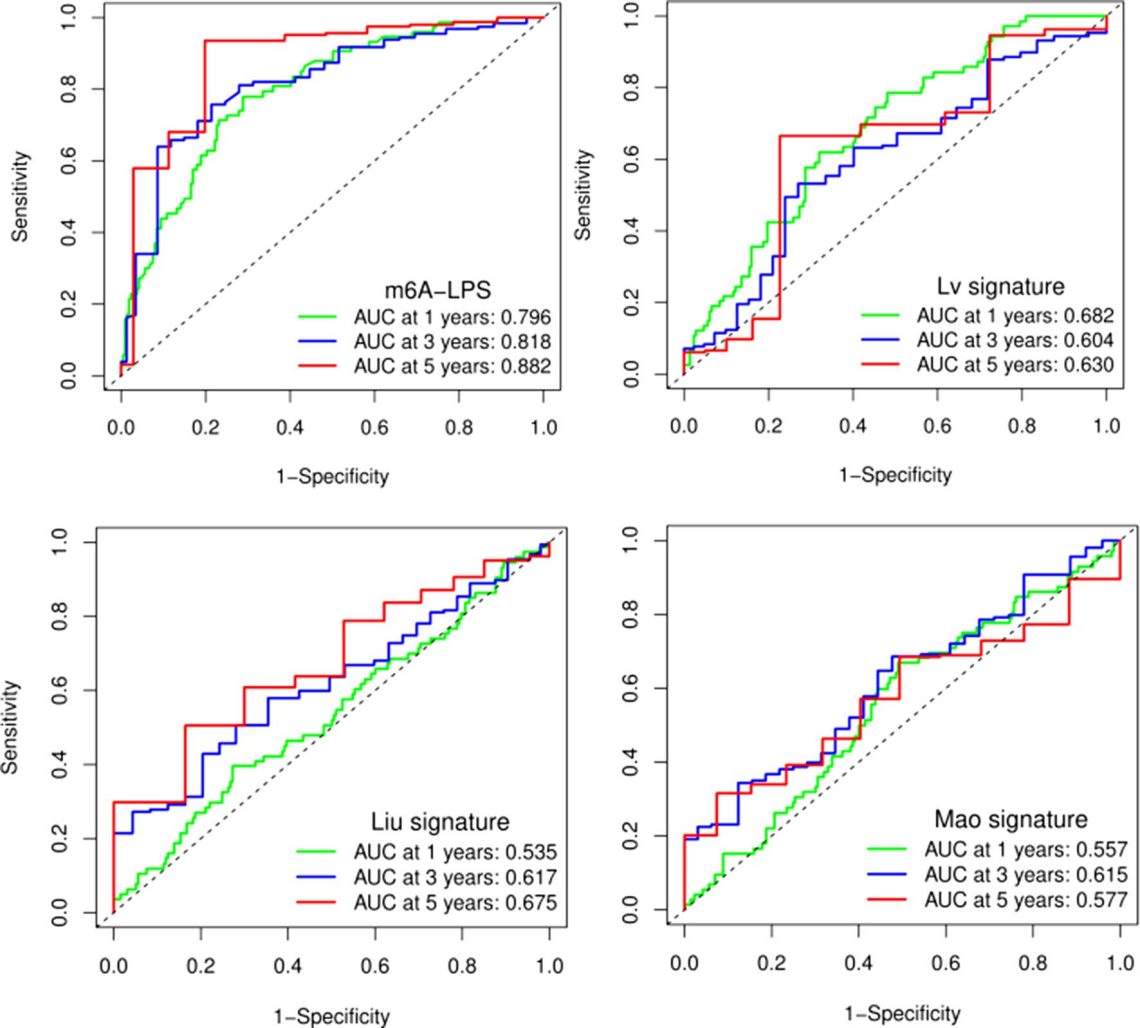
ROC analysis of different prognostic signatures. The 5-year overall survival AUC value of m6A-LPS model, Lv.signature model, Liu.signature model and Mao.signature model were 0.882, 0.630, 0.675 and 0.577, respectively

**Fig. 8:**
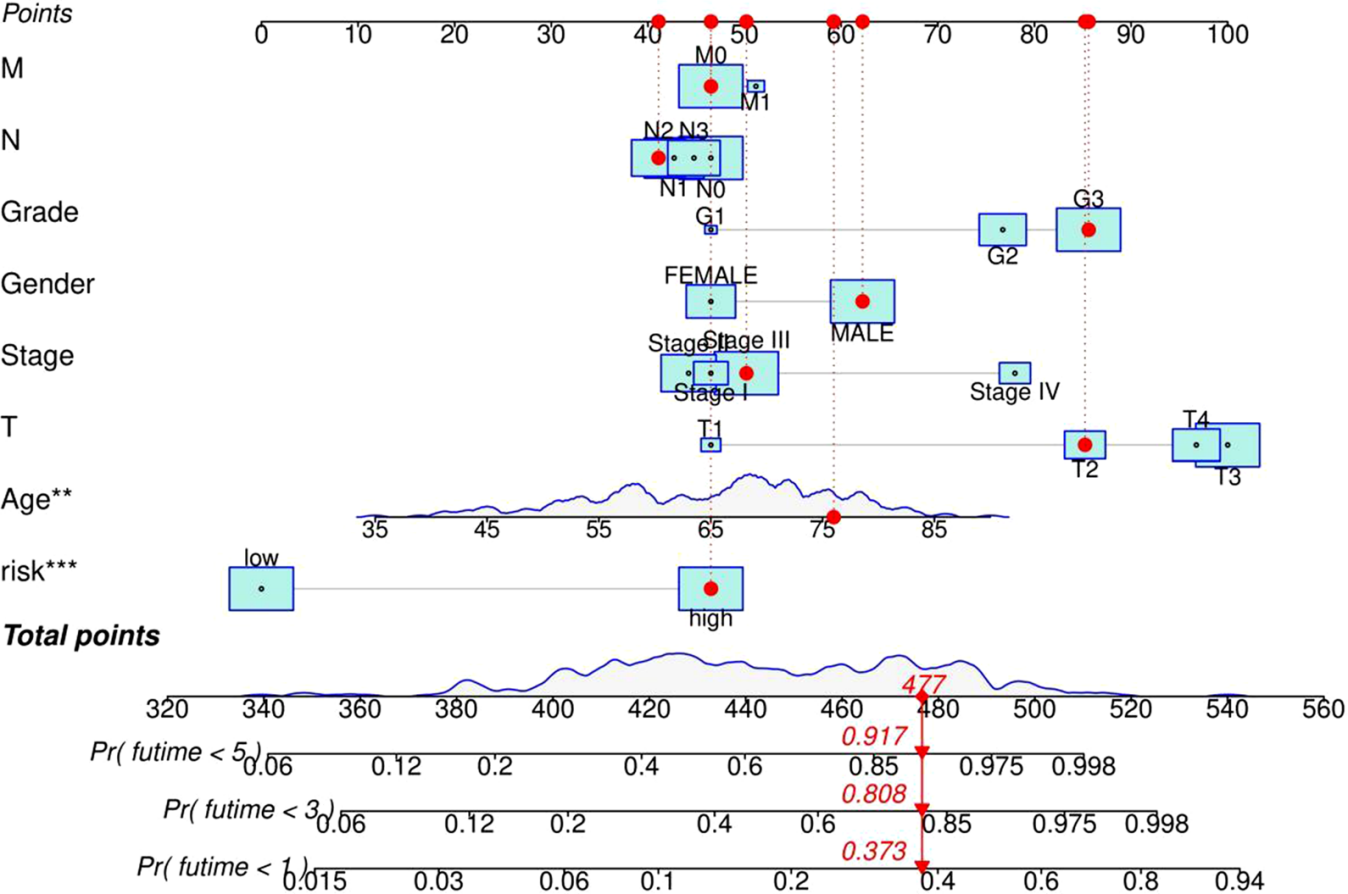
A nomogram including both clinicopathological factors and the m6A-LPS (**p < 0.05, ***p < 0.001)

The distribution of m6A-LPS was plotted along with the corresponding survival status based on the risk curve in Fig. 5a–c. The results showed that as the risk score increased, the number of deaths and the proportion of high-risk patients increased in the three datasets. Subsequently, to verify the clinical application value of the m6A-LPS, we performed univariate Cox regression analysis and multivariate Cox regression analysis of the m6A-LPS and clinicopathological characteristics, such as age, sex, grade and stage. The results revealed that m6A-LPS was an independent factor for predicting the prognosis of GC (*p* < 0.001) (Fig. 5d, e). Finally, to determine the predictive value of the m6A-LPS in different clinicopathological feature subgroups, we performed a stratified survival analysis. The survival curve revealed that m6A-LPS was a stable prognostic marker (*p* < 0.001) for GC patients stratified by age (<= 65 or > 65), sex (male or female), grade (G1-2 or G3), and stage (I–II or III–IV), as shown in Fig. 6. Furthermore, we compared our m6A-LPS with three published representative gene prognostic markers [23–25] using ROC curves for 1-, 3-, and 5-year OS, as shown in Fig. 7. The results showed that the 5-year AUC value of our prognostic model (the m6A-LPS) was 0.882, showing obviously higher predictive value and accuracy than the existing prognostic models Lv.signature (5-year AUC = 0.630), Liu.signature (5-year AUC = 0.675), and Mao. signature (5-year AUC = 0.577). Finally, the hybrid nomogram incorporating clinicopathological features and the m6A-LPS was also found to be stable and accurate, suggesting that it has potential value in the clinical management of GC patients (Fig. 8).

### GSEA

**Fig. 9:**
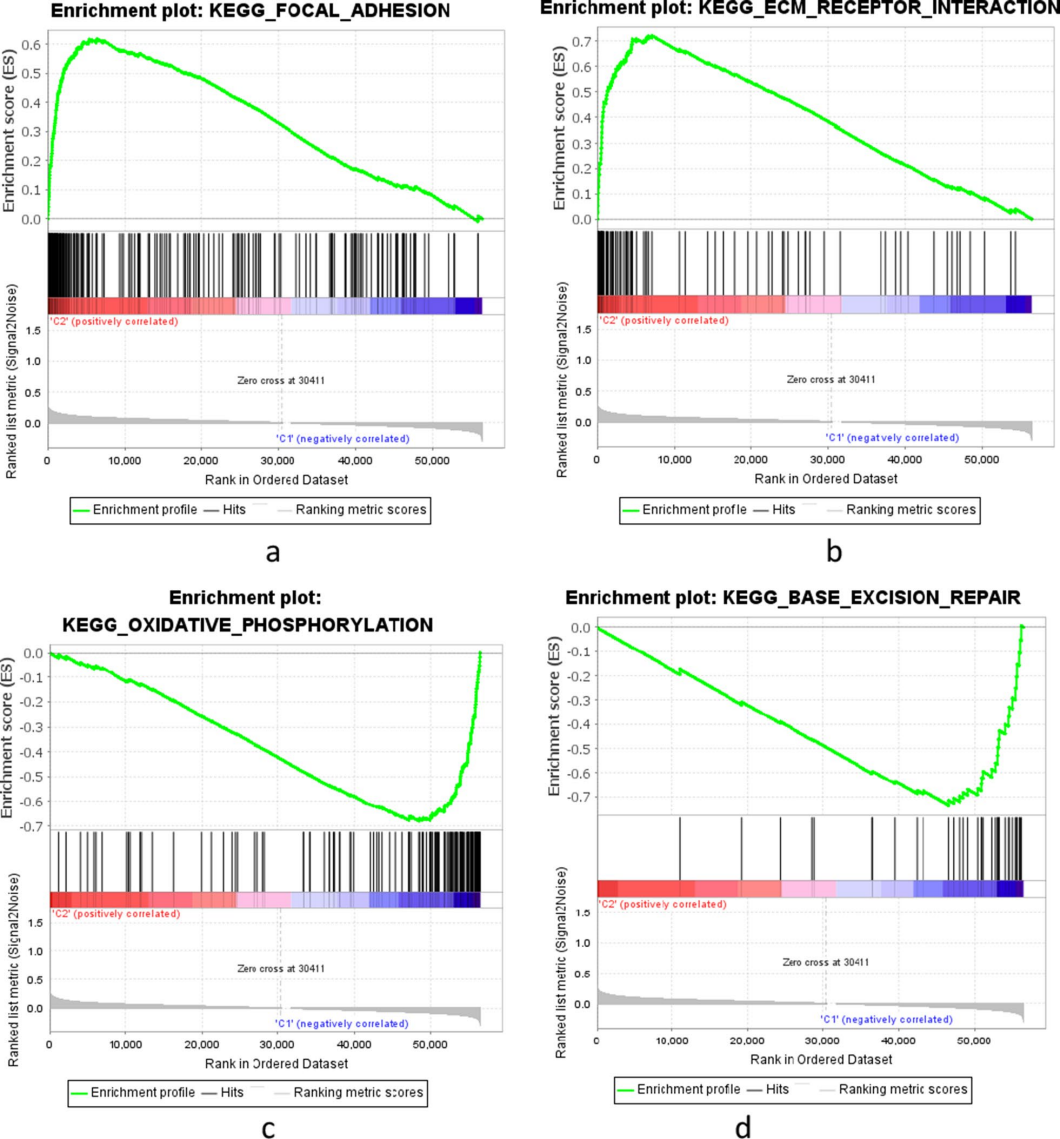
Gene set enrichment analysis of the m6A-LPS in the high-risk group (**a**, **b**) and low-risk group (**c**, **d**)

GSEA was used to explore the potential functions or pathways of the m6A-LPS. We defined the high-risk group as cluster 2 and the low-risk group as cluster 1. We found that patients in the high-risk group mainly showed enrichment of the terms extracellular matrix (ECM) receptor interactions and focal adhesion, while the low-risk group was characterized by enriched homologous recombination, oxidative phosphorylation and base excision repair (Fig. 9).

### Association between tumour-infiltrating immune cells and the prognostic model

**Fig. 10:**
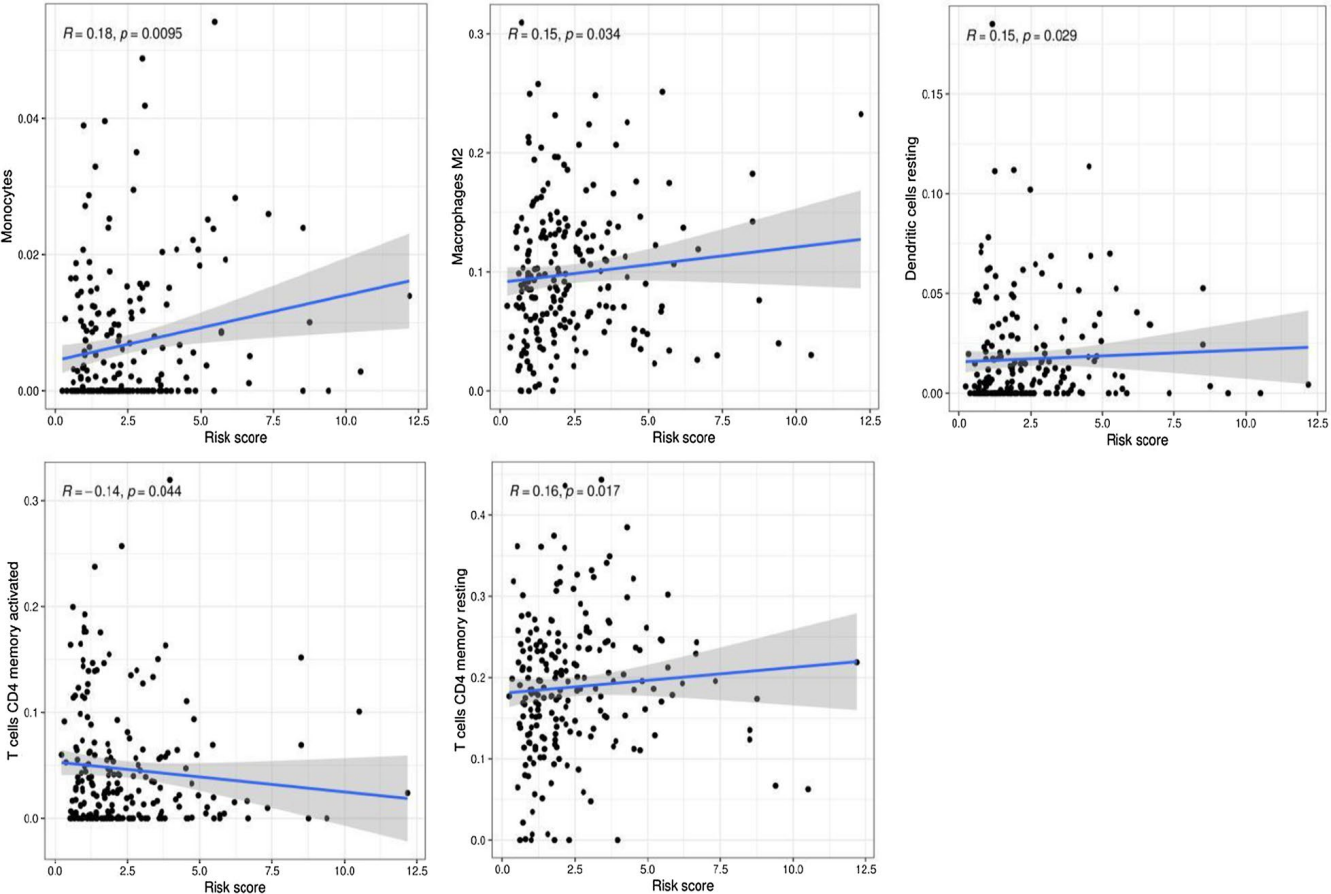
Potential relationships between the m6A-LPS and infiltrating immune cells

To explore the potential relationship between m6A-LPS and infiltrating immune cells, the Wilcoxon signed-rank test was utilized. The results revealed that monocytes (R = 0.18, *p* = 0.0095), M2 macrophages (R = 0.15, *p* = 0.034), resting dendritic cells (R = 0.15, *p* = 0.0029), and resting memory CD4 T cells (R = 0.16, *p* = 0.017) were positively correlated with the risk score, while activated memory CD4 T cells (R = − 0.14, *p* = 0.044) were inversely correlated with the risk score in the CIBERSORT dataset (Fig. 10).

### EMT biomarkers

**Fig. 11:**
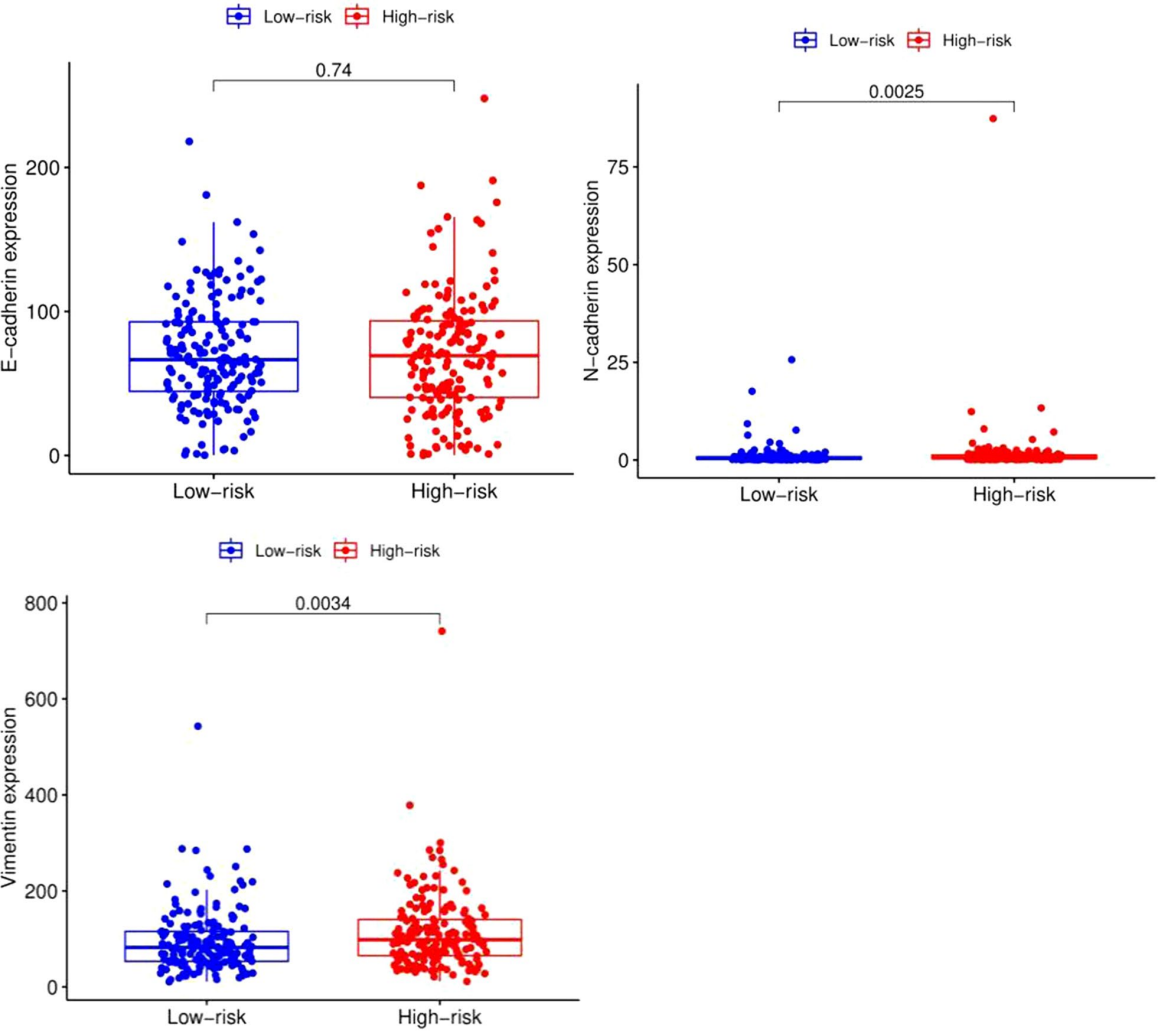
EMT biomarkers differentially expressed between the high-risk and low-risk groups

Increasing evidence has revealed that EMT is the basis of invasion and metastatic cancer cell spreading [20]. Therefore, we further determined EMT biomarkers that were differentially expressed in the high-risk and low-risk groups, and the results showed that *N*-cadherin and vimentin, which are markers of mesenchymal cells, were highly expressed in the high-risk group (*p* < 0.05); however, E-cadherin, a marker of epithelial cells, was not significantly different between the groups (*p* > 0.05) (Fig. 11).

## Discussion

With the rapid development of high-throughput sequencing and bioinformatics analyses, we are entering a new era of biological big data. A tremendous amount of genomic information, including potential biomarkers, can be detected in clinical samples, promoting the diagnosis, prognostication and prediction of diseases [26]. Genomic signatures are novel biomarkers in which genomic data are combined in a defined manner and have been proven to be able to predict the prognosis of patients with diseases, especially those with malignant tumours [27]. GC remains one of the most prevalent and deadly cancers worldwide, especially in China. Due to the lack of diagnostic biomarkers, most patients are diagnosed at an advanced stage, and not all patients benefit equally from surgical resection, chemotherapy or chemoradiotherapy because of disease heterogeneity [28]. In recent years, an increasing number of studies have focused on establishing signatures with both coding genes and noncoding RNAs to evaluate the prognosis of patients with cancer [29]. Several studies have revealed that m6A-related lncRNAs participate in the development of various cancers, including GC. Thus, exploring the role of lncRNAs in the prognosis and diagnosis of GC will contribute to a better understanding of the molecular mechanism of GC [13]. However, most prognostic signatures published to date [13, 23–25, 30, 31] require proper standardization of gene expression profile data for further analysis, which is a major limitation in clinical application. In the current study, we employed a strategy considering immune-related gene pairs [28] and attempted to construct an efficient model with two-lncRNA combinations regardless of exact expression levels [32]; this strategy not only eliminates batch effects among different platforms but also lacks the need for the normalization and scaling of data, thus successfully solving the problems surrounding the use of different data platforms to determine expression [33, 34].

First, raw lncRNA data were downloaded from the GC project of TCGA. By performing Pearson correlation coefficient analysis, iteration loop, 0-or-1 matrix, univariate Cox regression and LASSO-penalized regression analyses, we constructed an m6A-LPS (containing 14 m6A-related lncRNA pairs consisting of 25 unique lncRNAs). Based on the median risk score, patients were divided into high-risk and low-risk groups, and Kaplan–Meier curve analysis revealed that the high-risk group had shorter OS. Further ROC analysis revealed that the m6A-LPS had a higher accuracy in predicting the 5-year OS of GC than other traditional clinicopathological features. Moreover, multivariate Cox regression analysis revealed that m6A-LPS was an independent risk factor for GC. Notably, we also compared the accuracy of our model with that of other reported models. The AUC values of the Lv et al. seven-mRNA signature in predicting the 1-, 3-, and 5-year OS were 0.682, 0.603, and 0.630, respectively, and the AUC values of the Liu et al. four-gene signature in predicting the 1-, 3-, and 5-year OS were 0.535, 0.617, and 0.675, respectively. The AUC values of the Mao et al. six-gene signature in predicting the 1-, 3-, and 5-year OS were 0.557, 0.615, and 0.577, respectively, while the AUCs for our m6A-LPS model in predicting the OS at 1, 3, and 5 years were 0.795, 0.818, and 0.882, respectively. All of the above results demonstrate that our m6A-LPS provides efficient and robust prognostic prediction and might serve as an efficient biomarker for the prognosis of GC. In addition, a nomogram based on the m6A-LPS and clinicopathological factors may be applied in the clinical management of GC patients.

Furthermore, the GSEA results showed that patients in the high-risk group mainly showed enrichment of ECM receptor interactions and focal adhesion. Notably, previous studies have demonstrated that the ECM plays a vital role in cancer progression, and focal adhesion kinase (FAK) is often associated with poor clinical outcome, highlighting FAK as a potential determinant of tumour progression and metastasis [35]. The above results provide new directions for exploring the potential molecular mechanisms of GC.

Moreover, previous studies revealed that tumour-infiltrating immune cells can be used as independent prognostic markers in GC [36]. Therefore, we used CIBERSORT to explore the relationship between the risk score and tumour-infiltrating immune cells. The results showed that resting memory CD4 T cells, resting dendritic cells, monocytes, and M2 macrophages were positively related to the risk score, while activated memory CD4 T cells were inversely correlated with the risk score. Published studies have shown that increased monocytes and activated memory CD4 T cells are related to the poor prognosis of GC [37, 38], which is consistent with our research.

Finally, we also analysed the differential expression of EMT biomarkers between the high-risk and low-risk groups because the EMT process is a key molecular step in distant metastasis and is associated with poor prognosis [39]. The results showed that *N*-cadherin and vimentin, biomarkers of mesenchymal cells, were abundantly expressed in the high-risk group of patients. These results may provide new ideas for individualized treatment of GC patients.

Overall, we developed a prognostic model based on 14 m6A-related lncRNA pairs, and only the relative expression of the pairs had to be detected instead of examining specific expression values of every lncRNA, significantly lowering the cost of sequencing and carrying high clinical practicability. Furthermore, the prognostic model showed a robust, high value for predicting the survival of GC. However, this study has several limitations that need to be addressed. First, our prognostic model was constructed based only on TCGA data, and we failed to use other public databases or patient cohorts for further validation. Second, the relationship between m6A regulators and lncRNAs should be further explored in experiments in vitro and in vivo.

## Conclusion

In the current study, we constructed an m6A-LPS prognostic model with high predictive value that can serve as an independent prognostic factor for GC. To the best of our knowledge, this is the first study to construct a prognostic model based on m6A-related lncRNA pairs that does not require assessment of the exact expression levels of each lncRNA. Obviously, it has substantial value in clinical applications. Additionally, our results provide a new direction for individualized therapy.

## Data Availability

The raw data of this study are derived from the TCGA database (https://portal.gdc.cancer.gov/) and Kaplan–Meier Plotter data portal (http://kmplot.com/analysis/), which are publicly available databases.

## Abbreviations

m6A: N6-methyladenosine
GC: Gastric cancer
m6A-LPS: M6A-related lncRNA pairs signature
TCGA: The cancer genome atlas program
LASSO: The least absolute shrinkage and selection operator
ROC: The receiver operating characteristic curve
AUC: The area under the curve
DCA: The decision curve analysis
LncRNA: Long non coding RNA
EMT: Epithelial mesenchymal cell transition
HR: Hazard ratio
FC: Fold change
FDR: False discovery rate
OS: Overall survival
GSEA: Gene set enrichment analysis
KEGG: Kyoto enrcyclopedia of genes and genomes
KM: The Kaplan–Meier
ECM: Extracellular matrix
FAK: The focal adhesion kinase
